# The Neurophysiology of Language Processing Shapes the Evolution of Grammar: Evidence from Case Marking

**DOI:** 10.1371/journal.pone.0132819

**Published:** 2015-08-12

**Authors:** Balthasar Bickel, Alena Witzlack-Makarevich, Kamal K. Choudhary, Matthias Schlesewsky, Ina Bornkessel-Schlesewsky

**Affiliations:** 1 Department of Comparative Linguistics, University of Zürich, 8032 Zürich, Switzerland; 2 Department of General Linguistics, University of Kiel, 24098 Kiel, Germany; 3 Department of Humanities and Social Sciences, Indian Institute of Technology Ropar, Rupnagar 140001, India; 4 Department of English and Linguistics, University of Mainz, 55099 Mainz, Germany; 5 School of Psychology, Social Work and Social Policy, University of South Australia, Adelaide, 5001 South Australia, Australia; 6 Department of Germanic Linguistics, University of Marburg, 35032 Marburg, Germany; University of Edinburgh, UNITED KINGDOM

## Abstract

Do principles of language processing in the brain affect the way grammar evolves over time or is language change just a matter of socio-historical contingency? While the balance of evidence has been ambiguous and controversial, we identify here a neurophysiological constraint on the processing of language that has a systematic effect on the evolution of how noun phrases are marked by case (i.e. by such contrasts as between the English base form *she* and the object form *her*). In neurophysiological experiments across diverse languages we found that during processing, participants initially interpret the first base-form noun phrase they hear (e.g. *she*…) as an agent (which would fit a continuation like … *greeted him*), even when the sentence later requires the interpretation of a patient role (as in … *was greeted*). We show that this processing principle is also operative in Hindi, a language where initial base-form noun phrases most commonly denote patients because many agents receive a special case marker ("ergative") and are often left out in discourse. This finding suggests that the principle is species-wide and independent of the structural affordances of specific languages. As such, the principle favors the development and maintenance of case-marking systems that equate base-form cases with agents rather than with patients. We confirm this evolutionary bias by statistical analyses of phylogenetic signals in over 600 languages worldwide, controlling for confounding effects from language contact. Our findings suggest that at least one core property of grammar systematically adapts in its evolution to the neurophysiological conditions of the brain, independently of socio-historical factors. This opens up new avenues for understanding how specific properties of grammar have developed in tight interaction with the biological evolution of our species.

## Introduction

To what extent is linguistic structure shaped by universal properties of the brain or principles of communication, as opposed to culturally driven tendencies that happen to spread locally in specific regions or lineages? The balance of evidence is ambiguous. Several strands of research suggest that universal principles result in strong preferences for certain structures over others, e.g. regularities in syntax, sound systems or lexical structure [1–6]. However, many linguistic structures show highly distinctive geographical patterns that result from local spreads [7, 8], and there is conflicting evidence on the universality of some word order preferences [9–11].

Here, we provide evidence for a species-wide, culture-independent principle in a core area of grammar: the way in which argument roles of verbs are identified as agents or patients in sentences like *Ram* (agent) *sold a book* (patient). A key variable here is whether or not the A argument (the most agent-like argument) of transitive (multi-argument) verbs like *sell, see, hit* etc. is marked by a special identifier, conventionally called an ergative case marker [12–14]. English has no such marker, but languages like Hindi do. The sentence ‘Ram sold a book’ therefore translates into Hindi as *Rām-ne* (Ram) *kitāb* (book) *becī* (sold), with *Rām* marked by the ergative marker *ne*. Ergatives are limited to the A argument of transitive verbs, and thus set the A argument apart from the sole argument (S) of intransitive verbs like *sleep, work, slide* etc., which appear in the unmarked base form technically known as nominative or absolutive case (i.e. *Rām* without *ne* or any other such marker). Compare, for example, the ergative *Rām-ne* form in the transitive sentence *Rām-ne* (A) *kitāb becī* (‘Ram sold a book’) with the base form *Rām* in an intransitive sentence like *Rām* (S) *soyā* (‘Ram slept’). This contrasts with constructions like in English, where the S and A arguments are both in the base form, e.g. *he* in both *he* (S) *slept* and *he* (A) *sold a book*. This yields a grouping of S and A that corresponds to what is traditionally known as the subject function, a function that is not identifiable by case marking in ergative constructions [12, 14].

**Fig 1:**
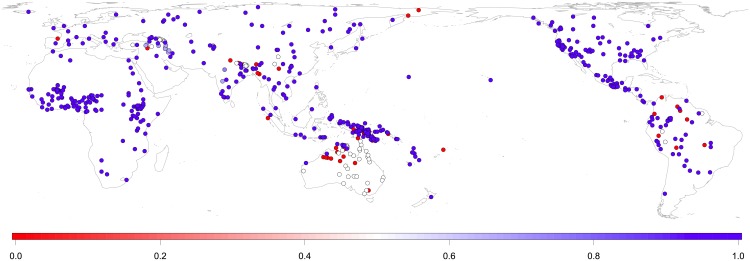
Ergative case marking. Some languages identify agents of transitive (multi-argument) verbs (e.g. *Ram* in *Ram sold a book*) by a special ‘ergative’ marker. The map shows the proportion of such markers per language (*N* = 617): 100% (red) means that the ergative occurs in all grammatical subsystems (e.g. all tenses, all persons), 50% (white) in half of all subsystems, 0% (blue) in none. Shades of blue represent ergative presence between 0% and 50%. Salient proportions of ergative markers are limited to the Pacific (New Guinea/Australia/Oceania) and South America regions.

An earlier survey of 194 languages suggests that ergative markers are universally disfavored in the languages of the world [15], i.e. languages seem to prefer case systems that treat S and A arguments alike over case systems that mark S and A differently. Our own database of 617 languages [Fig 1; S1 Database and S1 Sources] confirms this and also shows that substantial proportions of ergatives cluster only in what we call here the Pacific region (New Guinea, Australia, Oceania) and, to a lesser extent, in South America. Even in these regions, ergatives are often confined to subsystems of grammar, e.g. they occur only with third person pronouns, but not others, or only in certain tenses or aspects. In fact, the use of the Hindi ergative is also limited to sentences that contain what is called ‘perfective aspect’ verb forms.

**Fig 2:**
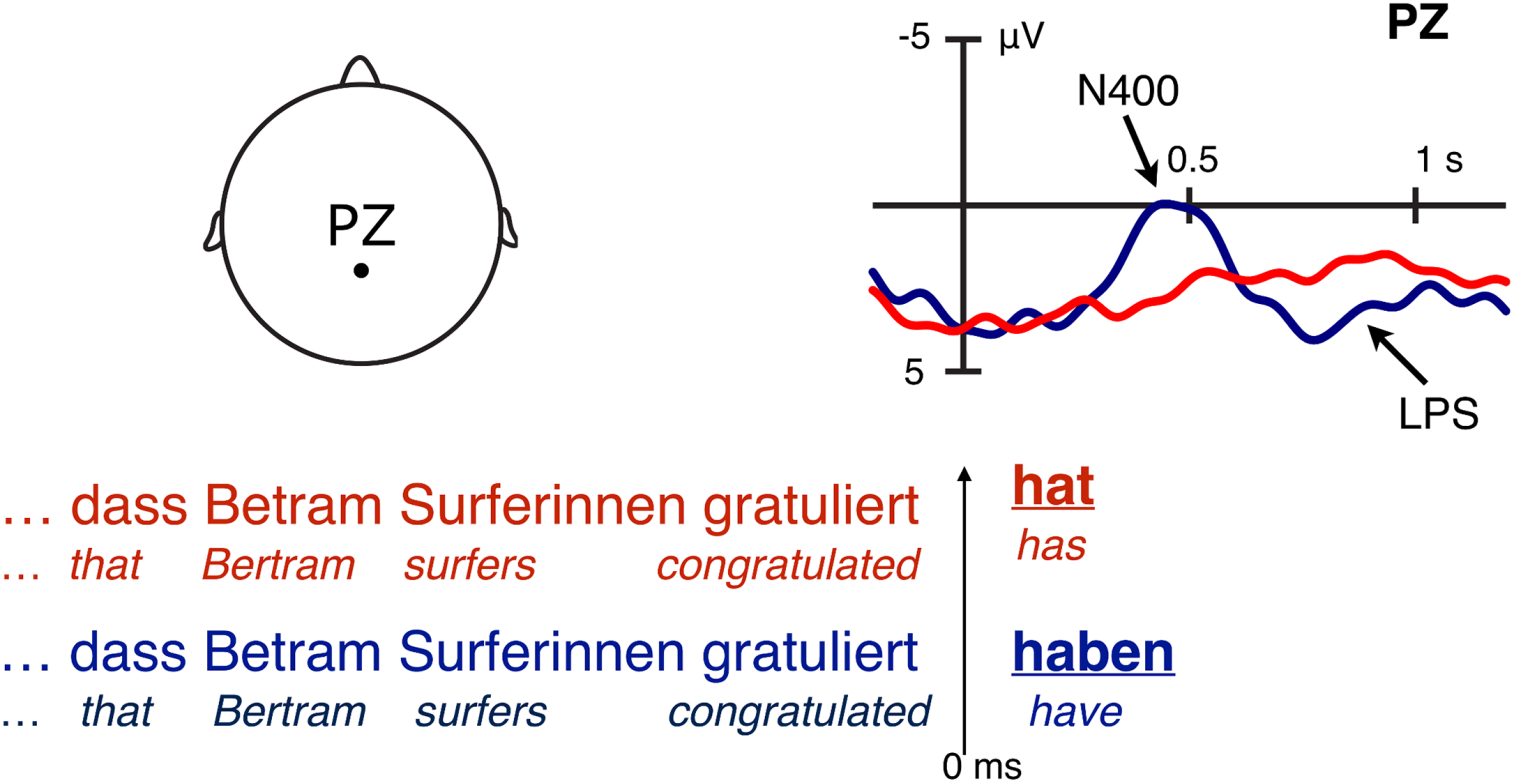
Event-related potentials (ERPs) at the end of a sentence in German. Before the final verb form (‘has’ vs. ‘have’) has entered the parsing system, the initial noun phrase argument (‘Bertram’) can be understood either as an agent (A) or as a patient (P). When the final verb form disambiguates the initial noun phrase towards a P reading (literally, ‘that the surfers have congratulated Bertram’), this triggers a biphasic N400—late positivity (LPS) pattern (blue trace). No pattern is observed when the final verb form disambiguates towards an A reading (‘that Bertram has congratulated the surfers’). This suggests that an A reading is the working assumption of the system right from the start [16], in line with the hypothesis of the S/A preference discussed in the main text. *Note:* Negativity is plotted upwards.

This skewed distribution raises the possibility that a species-wide principle acts against the development or maintenance of ergatives over time. A candidate for such a principle comes from neurophysiological research on language processing. When readers or listeners encounter a base-form noun phrase (NP) in a sentence (e.g. *Ram* or *a stone*), the processing system first assumes that this NP refers to the S argument (as in *Ram slept*) or to the A argument of a transitive verb (as in *Ram sold a book*). If processing the rest of the sentence falsifies this assumption (as in *Ram I sold a book*—a structure uncommon in English, but natural in other languages, e.g. German), one observes an event-related potential (ERP) signaling reanalysis of the role of the first NP, here as a patient (P) argument [Fig 2].

Typically, the ERP effect observed in this context is an N400—a centro-parietal negativity with a peak latency of approximately 400 ms—followed by a late parietal positivity (LPS) [16, 17]. The LPS is perhaps best known as occurring with a peak latency of approximately 600 ms (and is thus often referred to as a P600), but has also been reported in later time windows [18]. Both the N400 and the LPS have been observed in response to a variety of manipulations in language processing [19, 20]. In the context of word order and argument role disambiguation, however, extensive previous work on German has provided compelling evidence to suggest that the N400 effect observed in response to P-disambiguation indeed reflects reanalysis towards a dispreferred reading of the initial argument: For example, presence of an N400 disambiguation effect correlates with a decrease in processing speed (as measured by processing dynamics in the speed-accuracy tradeoff method and as predicted by the need for additional analysis steps), and N400 amplitude correlates with the ease or difficulty of computing the target structure required for correct interpretation [21]. Furthermore, no such effect is observed when the initial argument is marked as a P argument (e.g. by an accusative case marker), i.e. when no reanalysis is needed [22]. The LPS observed in these types of structures, by contrast, may reflect the task-dependent evaluation of the sentence being processed rather than reanalysis per se [16, 23].

This N400/LPS processing signal, which corresponds to the effects from what was known as a general subject preference in early behavioral work on Western European languages such as Dutch, German and Italian [24–29], has recently also been observed in a wider and more diverse range of languages, primarily using ERPs in simple sentences under various semantic and pragmatic conditions [17, 30]. In recognition of the fact that subjects are not identifiable by case markers in the presence of ergatives, we have coined the alternative, universally applicable label *S/A preference*: a preference to analyze an initial ambiguous base-form NP as bearing the S or A role [17, 31].

We assume that the S/A preference is primarily motivated by a fundamental principle of simplicity in processing [22, 32]: the simplest structure compatible with a base-form NP is one in which the NP is an S argument, as this entails no further dependency beyond the verb (i.e. an action or state of affairs with a single participant). If this fails (e.g. when there are more NPs and thereby more participants in the event described), the next simplest structure is one that assigns the initial base-form NP the agent (A) role. This structure is the next simplest because patients (P arguments) causally and existentially presuppose agents while the reverse is not true [33]: if a patient is affected by a causal action, there must be someone or something, i.e. an agent, instigating that action. But an agent can carry out actions without a patient, as when we say, for example, that someone was working all day. Therefore, patients create more, and more complex dependencies between participants than agents. Conversely, an early and privileged assignment of agents is consistent with the finding that agents are the point of departure for the cognitive construction of action in general, also outside language—possibly because this type of event construction became hard-wired in the evolutionary history of our brains [34].

This theory predicts that the S/A preference is a species-wide property of the processing system, independent of the structural affordances of specific languages, and also independent of specific contexts of language use. This prediction is borne out to the extent that the S/A preference has been replicated in a variety of languages and under varied contextual conditions, such as the animacy and topicality of NPs and the frequency of patient-initial sentences in language use [17, 30].

If the S/A preference is therefore truly universal, it would explain the observation in Fig 1, i.e. that languages prefer base forms to treat S and A alike rather than to set apart these roles by using an ergative marker on the A argument. If case marking has or prefers base-form NPs which do not group S and A, the S/A preference will systematically lead to a reanalysis effect whenever a base-form NP is in initial position and the sentence ends in a transitive verb: the processing system will automatically first assign an S or A interpretation but will then need to revise this assignment whenever the verb suggests that the first NP was not an S or an A argument, but, for example, a P argument. This systematic reanalysis costs extra energy. The costs cannot be so high that the language cannot be processed anymore, since ergatives are attested and can be as easily transmitted across generations as any other case marker [35]. However, assuming that the brain tends to avoid extra costs and therefore optimizes language structures so that they fit best with its needs [2, 4, 36], one would predict that the S/A-preference would repeatedly prompt users of ergative case marking systems to restructure their grammar as one that lacks an ergative, so that base-form NPs treat S and A alike.

Such a change can be actuated at any time, and with any language user. Whether or not the change becomes an established feature of the grammar, however, depends on whether or not the new structure catches on in the speech community [37]. This is a social process, subject to many counter-acting factors, such as conservatism (why change a pattern if it works?), populism (keeping ergatives if everyone else does), or independent motivations for the maintenance or even new developments of ergatives (e.g. as markers for highlighting the special saliency of agents [13], or from nominalizations [38] and participial tense constructions [39–41] with involved participants in an oblique case that eventually develop into ergative A arguments). But if the S/A-preference is strong enough, one would nevertheless expect it to shape language evolution often enough to leave a detectable statistical signal.

This scenario makes two predictions, both of which we will test in the following. First, if the S/A preference is truly universal, it should be detectable even in languages with ergative case marking, i.e. we expect the processing system not to adapt to the special affordances of such languages but to constantly produce the same small reanalysis effects that have been observed for languages without ergatives, like German [Fig 2]. We test this here with an electrophysiological experiment on the comprehension of initial NPs in Hindi. Hindi is a suitable test case not only because of the availability of ergative case marking but also because initial NPs are in fact most commonly used to denote P arguments in actual language use, a pattern that reflects a tendency to drop S and A arguments because they are often topical [42]. We will focus specifically on inanimate NPs (like ‘book’, ‘stone’ etc.) because these are the least likely to be used as agents. Therefore, if the processing system transiently assigns an agent role to even these NPs, this would support the proposal that the S/A preference is indeed an automatic and robust property of the system. In a previous study, we found a processing preference for verb-agreement with S or A arguments in Hindi in spite of the fact that, depending on several conditions, S, A and P arguments can all trigger verb agreement in this language [31]. While this observation lends initial support to the assumption that S/A alignments are favored during the online comprehension of Hindi, no study to date has investigated whether this preference extends to role assignment in initial NPs.

Second, if the brain tends to optimize grammars so that they fit the needs of processing preferences, we should find that in their historical evolution, languages are more likely to maintain and develop non-ergative case-marking systems (treating S and A alike) than ergative case-marking systems (splitting S and A). We test this here by estimating the extent to which language families (groups of languages of shared descent, such as Indo-European or Austronesian, but also single-member units, i.e. languages with no known surviving relatives, such as Basque) have undergone a probabilistic bias towards or against ergatives during their phylogenetic evolution. Given the uneven distribution of ergatives in Fig 1, we will control for possible confounds resulting from the local spread of case systems through language contact.

Note that both predictions only apply to languages which regularly have initial base-form NPs. The S/A preference is not necessarily expected to affect verb-initial languages since, after processing the initial verb, dependencies are already established at the outset, making reanalyses of NP roles unlikely [43, 44]. Therefore, the S/A preference makes no prediction on the evolution of case-marking system in these languages either, and so our study excludes verb-initial languages (which make up only less than 10% of the world’s known languages [45]).

## Evidence from Neurophysiology

### Materials and Methods

#### Participants

Thirty-two right-handed native speakers of Hindi (6 women, mean age: 26.13) participated in the experiment. Participants had normal or corrected-to-normal vision and no history of neurological conditions. The majority of participants were from the Indian states of Uttar Pradesh and Madhya Pradesh. Data acquisition took place at the University of Mainz, Germany. All participants had learnt Hindi before the age of six, but also spoke other languages, mainly other Indian languages, English, and in a few cases also German (non-fluently). (Due to the language plurality in India, monolingual native speakers of Hindi are rare.)

#### Ethics statement

The experiment was performed in accordance with the ethical standards laid down in the Declaration of Helsinki. Approval by an ethics review board was not required, as current regulations in Germany specify that ethics approval for ERP experiments is only required when participants are patients, children or older adults (> 65 years) [46]. Participants, who were native speakers of Hindi residing in Mainz, Frankfurt, Darmstadt and surrounding areas, were recruited specifically for this study by word of mouth, posts on internet forums, social media and flyers. Demographic data collected from participants (name, age, gender, handedness, languages spoken, level of education) was stored separately from the results collected as part of the experiment (error rates, reaction times and EEG responses), i.e. experimental results were processed in anonymized form. Participants gave written informed consent before the beginning of the experiment and were informed that they could discontinue the study at any time should they wish to do so.

#### Data availability

The raw behavioural and EEG data from this experiment are publicly accessible via http://figshare.com (http://dx.doi.org/10.6084/m9.figshare.1394600).

#### Materials

The sentence materials comprised 60 sets of the conditions illustrated in Table 1 and S1 Table. All four sentences in a set expressed the same proposition (e.g. Gopal selling a book) in a P-Verb-A word order (‘book sell Gopal’), with the following variations. Firstly, the initial NP (*kitāb* ‘book’) was either left in the base form (‘nominative’, ambiguous conditions AI and AP) or marked with the accusative marker *-ko* (unambiguous conditions UI and UP). Accusative case marking, which unambiguously identifies the initial inanimate NP (*kitāb-ko*) as a P argument, is optional for inanimates in Hindi in that it is only used to mark definite or specific entities (i.e. while *kitāb* refers to ‘a book’, *kitāb-ko* refers to ‘the book’ or ‘a particular book’). Thus, while the initial NP was initially ambiguous between an S, A and P reading in the AI and AP conditions and only disambiguated as a P argument at the position of the verb, the initial NP in the UI and UP conditions was immediately recognizable as a P argument. Hence, the UI and UP conditions served as controls for the AI and AP conditions at the position of the verb, i.e. by contrasting A and U conditions, we were able to isolate the effect of disambiguating the initial NP towards a P reading, while keeping overall clausal word order and propositional meaning identical. The second manipulation involved the contrast between imperfective (AI/UI) and perfective aspect (AP/UP). This manipulation was included as Hindi only uses ergative case marking in the perfective aspect. We thus aimed to examine whether the effects of disambiguating an initial base-form NP towards a P reading would be influenced by the aspect-dependent availability of an ergative marker.

**Table 1:** Sample set of materials from the electrophysiological experiment on Hindi (with masculine gender A arguments; for feminine gender examples, see S1 Table).

Condition	Examples				Mean accuracy
AI	*kitāb*	*beca-tā*	*hai*	*Gopāl*	94 (5)
	book(F)[NOM]	sell-I.M	AUX	Gopal(M)[NOM]	
	‘Gopal sells a book.’				
UI	*kitāb-ko*	*beca-tā*	*hai*	*Gopāl*	92 (7)
	book(F)-ACC	sell-I.M	AUX	Gopal(M)[NOM]	
	‘Gopal sells the book.’				
AP	*kitāb*	*bec-ī*	*hai*	*Gopāl-ne*	95 (4)
	book(F)[NOM]	sell-P.F	AUX	Gopal(M)-ERG	
	‘Gopal has sold a book.’				
UP	*kitāb-ko*	*bec-ā*	*hai*	*Gopāl-ne*	94 (6)
	book(F)-ACC	sell-P.M	AUX	Gopal(M)-ERG	
	‘Gopal has sold the book.’				

Mean accuracy reports the accuracy for comprehension questions in %, with standard deviations by participants in brackets. *Condition codes:* A, ambiguous; U, unambiguous; I, imperfective aspect (not triggering ergative marking); P, perfective (triggering ergative marking). *Glosses:* AUX, auxiliary; ACC, accusative; ERG, ergative; F, feminine; M, masculine; NOM, nominative.

The first NP in critical sentences was always inanimate. This pushes the meaning context against our hypothesis, strengthening possible results. In addition, inanimates allow the manipulation of accusative case marking as described above, while animates invariably require accusative marking. All verbs in the stimulus sets were transitive. The sentence-final A-argument was always a proper name. In order to counterbalance gender of the arguments, the first 30 sentence sets contained feminine P arguments and masculine A arguments (as illustrated in Table 1), while gender assignments were reversed in the remaining sets (as illustrated in S1 Table).

The 240 critical sentences (60 sets of lexical material in four conditions) were subdivided into two lists of 120 sentences each (2 from each of the 60 lexical sets and 30 per condition). Each list was pseudo-randomly interspersed with 240 filler sentences of various types, such that each participant read 360 sentences in total. Note that the filler sentences included S- and A-initial sentences with base-form initial arguments in order to ensure that participants could not anticipate the fact that the initial NPs in the critical sentences were always P arguments.

#### Procedure

The sentences were presented visually in Devanagari script and word-by-word (nouns and case markers written together) in the centre of a computer screen with a presentation time of 650 ms per word and an inter-stimulus-interval (ISI) of 100 ms. Each trial began with the presentation of an asterisk (1000 ms plus 200 ms ISI) and ended with a 1000 ms pause, followed by a comprehension question for assessing whether participants processed the stimuli attentively and accurately. Participants responded to the question by pushing buttons for ‘yes’ or ‘no’ on a hand-held game controller. Assignment of the left and right buttons to ‘yes’ and ‘no’ answers was counterbalanced across participants. The questions rephrased the sentence that the participants had read and were correct in 50% of all trials. Incorrect questions were created by exchanging NP1 and NP2 or the verb. The maximal response time for the comprehension question was set at 4000 ms, after which participants were notified that the answer time had expired and the next trial was initiated. The inter-trial interval was 2000 ms.

Participants were asked to avoid movements and blinking while reading the sentence, but were allowed to blink while reading the comprehension questions. An additional practice session (20 sentences) was conducted for each participant before the main experiment started in order to familiarize participants with the procedure and the task. In the main experiment, the 360 sentences were presented in 9 randomized blocks of 40 sentences each. There was a short break after each block. Including electrode preparation, the entire experiment lasted approximately 3 hours.

#### EEG recording and preprocessing

The EEG was recorded from the following 25 AgAgCl electrodes positioned according to the extended 10–20 system (American Electroencephalographic Society, 1994) using an elastic cap (Easy Cap GmbH, Herrsching, Germany): F7, F3, FZ, F4, F8, FC5, FC1, FCZ, FC2, FC6, CZ, CP5, CP1, CPZ, CP2, CP6, P7, P3, PZ, P4, P8, POZ, O1 and O2. AFZ served as ground. Recordings were referenced to the left mastoid, but re-referenced to linked mastoids offline. The electrooculogram (EOG) was monitored by means of electrodes placed at the outer canthi of a participant’s eyes and above and below his/her right eye. All EEG and EOG channels were amplified using a BrainAmp amplifier (Brain Products GmbH, Gilching, Germany) and digitized with a sampling rate of 500 Hz. In order to exclude slow signal drifts, raw data were filtered with a 0.3–20 Hz bandpass filter offline. ERP plots were additionally smoothed using an 8 Hz lowpass filter.

#### Data analysis

For the behavioral data (accuracy rates for the comprehension questions), a repeated measures analysis of variance (ANOVA) was performed with the condition factors ambiguity (NP1 ambiguous vs. NP1 marked as a P argument), aspect (imperfective vs. perfective), and gender (masculine vs. feminine P argument), and the random factors participants (*F*_1_) and items (*F*_2_). Note that the factor gender, which was included in order to examine possible differences between the sentence sets with different gender assignments to A and P arguments (see Materials section), was manipulated within participants and between items and thus modelled differently in the analyses by participants and items. We refrained from analyzing reaction times as reactions were delayed relative to the critical manipulation.

Average ERPs were calculated per condition, participant and electrode from -200 to 1200 ms relative to the onset of the critical verb position for all artifact-free trials. Artifacts were identified via both automatic and manual scanning procedures (the criterion for automatic EOG artifact identification was a change in 40 V in a sliding window of 20 ms). Subsequently, grand-averages were computed over all participants. Repeated measures ANOVAs were performed with the within-participant factors ambiguity, aspect, and gender and the topographical factor Region of Interest (ROI). Lateral and midline sites were analyzed separately, with regions of interest defined for the lateral electrodes as follows: left-anterior (F7/F3/FC5/FC1), left-posterior (P7/P3/CP5/CP1), right-anterior (F8/F4/FC6/FC2), and right-posterior (P8/P4/CP6/CP2). For the midline electrodes, each electrode was analyzed as a ROI of its own: FZ, FCZ, CZ, CPZ, PZ and POZ. In order to avoid Type I errors due to violations of sphericity, we report Huynh-Feld-corrected *p*-values for all effects with more than 1 degree of freedom in the numerator and for which Mauchley’s test for sphericity reached significance. The statistical analysis was performed using the ez-package [47] in R [48].

### Results

#### Behavioural data

Mean accuracy rates for the comprehension questions are included in Table 1. As evidenced by > 90% accuracy in responses to comprehension questions for all conditions, participants processed all sentences attentively and accurately, including those requiring a reanalysis of the role of the first NP as being a P argument. A repeated measures ANOVA for the accuracy rates revealed a main effect of ambiguity, which, however, only reached significance in the analysis by participants (*F*_1_(1, 31) = 4.87, *p* < .04; *F*_2_(1, 58) = 2.91, *p* > .09). No other effects reached significance (all *F*s < 1, except for a main effect of aspect with *F*_1_(1, 31) = 2.97, *p* > .09 and *F*_2_(1, 58) = 1.74, *p* > .19, a main effect of gender with *F*_1_(1, 31) = 1.45, *p* > .23 and *F*_2_ < 1, and an interaction of aspect × gender with *F*_1_(1, 31) = 3.19, *p* > .08 and *F*_2_(1, 58) = 1.21, *p* > .27).

#### ERP data

The S/A preference hypothesis predicts that, even in a (split-)ergative language such as Hindi, initial arguments are transiently analyzed as S or A arguments as long as they are not unambiguously marked as a P argument. We thus expect to observe a reanalysis (or surprisal) effect when this assumed reading does not hold. In the context of the present design, this led us to predict an effect of ambiguity (P-disambiguation) at the position of the disambiguating verb. We further examined whether this effect is modulated by aspect (perfective vs. imperfective), i.e. the presence vs. absence of ergative case marking. This additional modulation allowed us to examine whether the online analysis of the initial argument was driven primarily by a preference for NP1 to trigger verb agreement, since this preference is upheld in the perfective conditions (which require agreement with the P argument when it is in the base form) but not in the imperfective conditions (which require agreement with the S or A argument). An agreement-driven analysis strategy of the initial argument should therefore be expected to engender an interaction between ambiguity and aspect, while an analysis based on a preference for an S or A reading of NP1 should manifest itself in a main effect of ambiguity. These competing hypotheses were tested by means of repeated-measures ANOVAs at the position of the critical disambiguating verb, which we report below.

**Fig 3:**
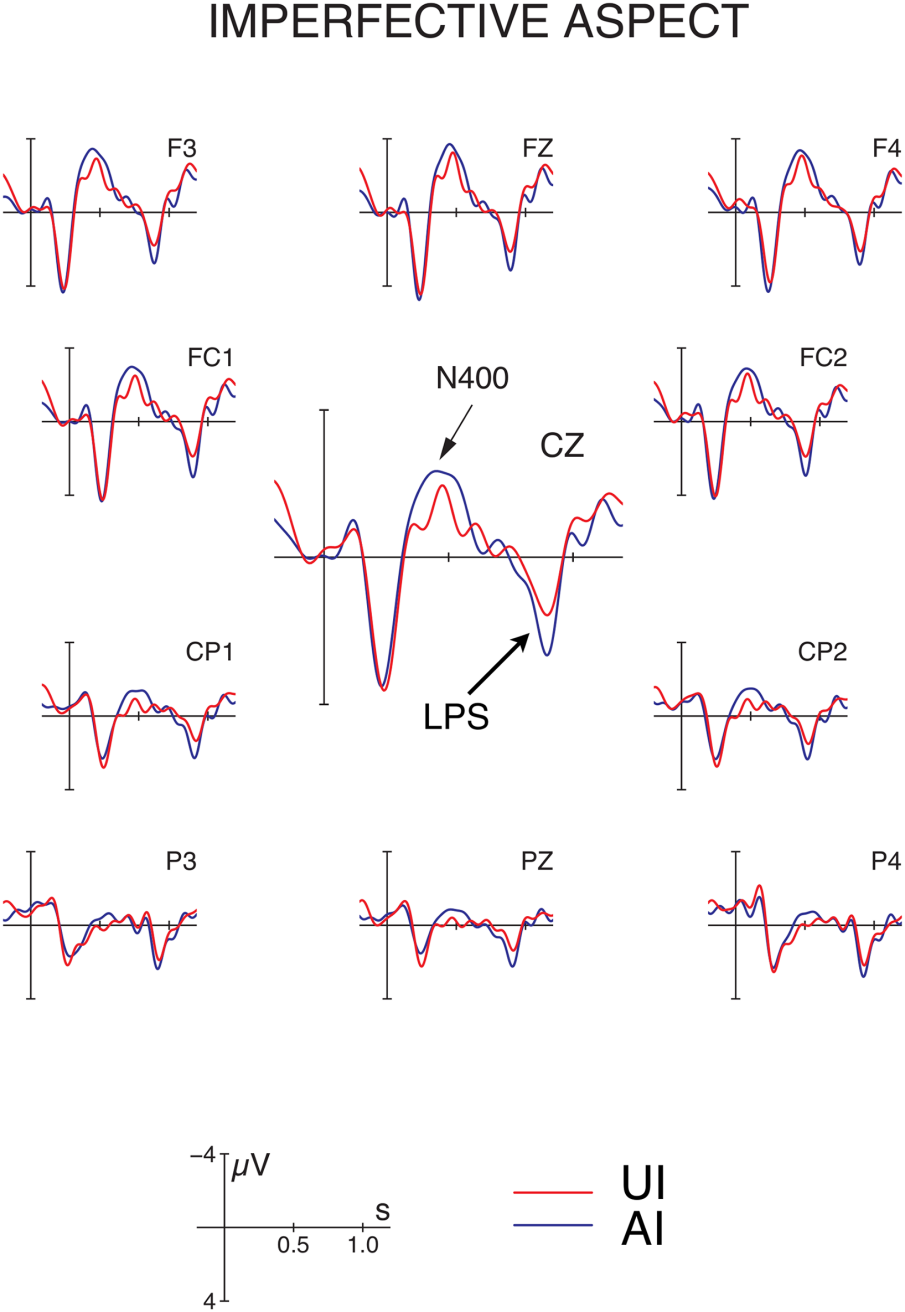
Event-related potentials (ERPs) at the position of the disambiguating verb in the imperfective conditions in Hindi. The figure shows grand average ERPs following a locally ambiguous (blue trace) or unambiguous (red trace) P argument at 11 selected electrodes. See Table 1 for examples. Disambiguation of an initial base-form (ambiguous) NP towards a P argument engenders an N400—late positivity (LPS) response, consistent with the S/A preference. *Note:* Negativity is plotted upwards.

**Fig 4:**
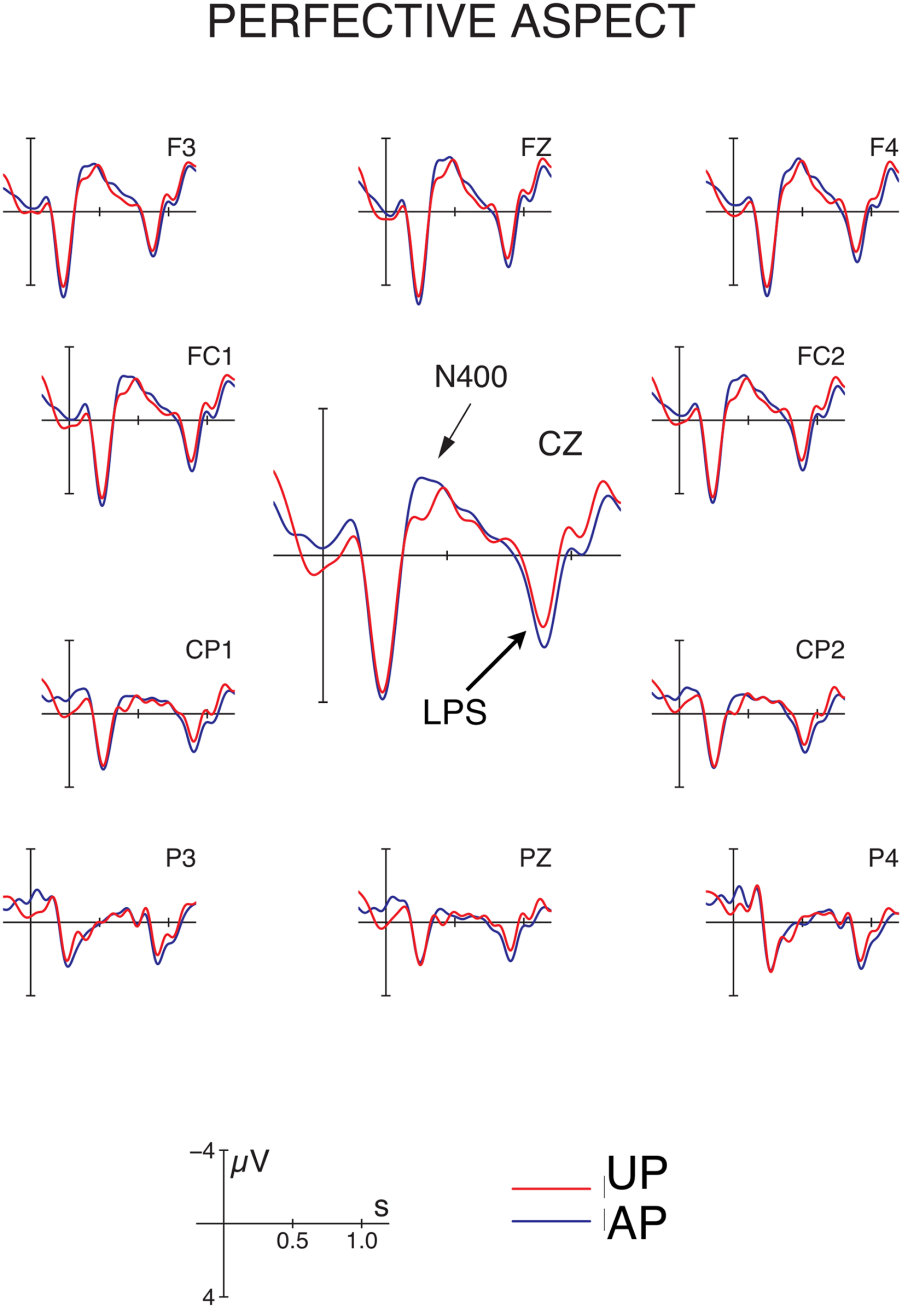
Event-related potentials (ERPs) at the position of the disambiguating verb in the perfective conditions in Hindi. The figure shows grand average ERPs following a locally ambiguous (blue trace) or unambiguous (red trace) P argument at 11 selected electrodes. See Table 1 for examples. Disambiguation of an initial base-form (ambiguous) NP towards a P argument engenders an N400—late positivity (LPS) response, consistent with the S/A preference. *Note:* Negativity is plotted upwards.

Grand average ERPs time-locked to the position of the critical verb are depicted in Figs 3 and 4 for imperfective and perfective conditions, respectively. Visual inspection of the figures suggests that the conditions with base-form initial P arguments (AI and AP) elicited a biphasic N400—late positivity (LPS) response in comparison to the unambiguously marked controls (UI and UP). For statistical analysis of the data, we selected the following time windows based on visual inspection: 300–550 ms (N400) and 750–900 ms (LPS). Below we only report effects involving the factor ambiguity since main effects of aspect or interactions of aspect and ROI are not relevant to the research question under examination here.

*N400 time window (300–550 ms)*: For the N400 time window, repeated measures ANOVAs revealed main effects of P-disambiguation (effect of ambiguity: lateral electrodes: *F*(1, 31) = 12.08, *p* < .002; midline electrodes: *F*(1, 31) = 15.62, *p* < .0005), due to more negative-going ERPs for ambiguous versus unambiguous conditions. For the midline electrodes, this effect was qualified by an interaction with the topographical factor Region of Interest (*F*(5, 155) = 6.77, *p* < .007), revealing a central distribution (effects of P-disambiguation at FZ, FCZ, CZ and CPZ; all *p*s < .01). For lateral electrode sites only, we further observed a main effect of aspect (*F*(1, 31) = 4.80, *p* < .004), which resulted from more negative ERP amplitudes for imperfective vs. perfective sentences, and a (non-significant) trend towards an aspect × ambiguity interaction (*F*(1, 31) = 3.60, *p* < .07). Neither of these effects approached significance at midline sites (aspect: *F*(1, 31) = 1.22, *p* > .27; aspect × ambiguity: *F*(1, 31) = 2.04, *p* > .16).

*LPS time window (750–900 ms)*: The analysis of the LPS time window also revealed main effects of ambiguity, with a marginal effect of P-disambiguation at lateral electrodes (*F*(1, 31) = 4.14, *p* = .05) and a significant effect at midline sites (*F*(1, 31) = 10.16, *p* < .004). Both analyses further showed a significant main effect of gender (lateral electrodes: *F*(1, 31) = 11.90, *p* < .002; midline electrodes: *F*(1, 31) = 12.36, *p* < .002), which was due to more positive-going ERPs for sentences with masculine vs. feminine initial P arguments. No interactions approached significance in this time window.

In summary, disambiguation of an initial NP towards a P reading engendered a biphasic N400—LPS response in Hindi, which was observable in both imperfective and perfective aspect.

### Discussion

The electrophysiological findings provide evidence for an S/A preference irrespective of aspect, i.e. the N400-LPS pattern for disambiguation towards a P-reading of the initial argument was observable in both imperfective and perfective aspects. Observing an effect of P-disambiguation irrespective of aspect suggests that the effect cannot be reduced to a preference for verb agreement between the initial NP and the verb, since agreement in Hindi depends on aspect: verbs agree with a base-form P or S argument in the perfective but with the base-form A or S argument in the imperfective aspect. While the ambiguity × aspect interaction did show a trend towards significance at lateral sites in the N400 time window, reflecting a numerically larger disambiguation effect for imperfective vs. perfective sentences, there was no hint of an ambiguity × aspect interaction at midline sites in the N400 window or in any analysis for the LPS window. Thus, while the additional agreement mismatch in the imperfective conditions may have contributed in part to the—numerically—larger N400 disambiguation effect at lateral sites, both perfective and imperfective conditions show a clear S/A preference as evidence by the robust, across-the-board occurrence of the N400—LPS disambiguation effect.

Prima-facie alternative interpretations of our findings include the possibility that the N400-LPS pattern at the verb reflects a general surprisal effect [49] due to verbs being more expected after *ko*-marked as opposed to unmarked base-form arguments, or an effect reflecting thematic processing due to an underspecification of thematic roles prior to the verb for base-form (nominative) but not for case-marked (accusative or ergative) arguments. Both possibilities appear unlikely in view of our previous results on agreement processing in Hindi [31]. Firstly, our previous study indicated that there is a clear processing preference for an initial base-form NP to trigger verb agreement. In this way, the verb should engender a higher degree of surprisal when this condition is not met in comparison to when agreement is present. A surprisal-based perspective would thus predict a significantly higher ERP effect in condition AI (in which the initial NP does not trigger agreement) compared to AP (in which it does) at the position of the verb. However, as noted above, no such interaction was significant in our results.

With respect to the second alternative interpretation, namely thematic underspecification of ambiguous NPs, note that our previous study [31] compared sentences beginning with two base-form arguments with sentences beginning with an ergative-marked and a base-form argument. Assuming a thematic underspecification of base-form NPs—with a subsequent assignment of thematic roles at the verb engendering an N400—LPS pattern—we should have observed a similar ambiguity effect in our previous experiment, which, however, was not the case (note that the initial argument was always an A argument in that study). Taken together, the results of the present study and the experiment reported in [31] thus suggest that the effects observed here are specific to a situation in which an ambiguous initial NP is disambiguated towards a P reading, thereby supporting our interpretation in terms of reanalysis rather than alternative possibilities.

A potential limitation of our findings comes from the fact that we tested the S/A preference hypothesis using a language that has both ergative and non-ergative case-marking structures. In view of this split, it appears possible that Hindi may behave just like a language without ergatives early in the sentence, when aspect is still ambiguous. If this were the case, it would mean that, by default, the Hindi processing system assumes a non-ergative case-marking structure when there is no evidence to the contrary. This would predict an effect of aspect at our critical verb position, reflecting increased surprisal or reanalysis for the perfective, ergative-licensing conditions. Contrary to this prediction, however, we observed an increased (rather then decreased) negativity for verbs with imperfective as opposed to perfective aspect conditions in the N400 time window and no effect of aspect at all in the LPS time window. Thus, while there is evidence to suggest that Hindi speakers establish predictions about the aspect of a clause even before the verb is encountered, based on a variety of morphological and semantic cues [50, 51], there does not appear to be a default preference for imperfective and non-ergative case-marking structures. This suggests that both ergative and non-ergative structures are considered as viable options during incremental sentence comprehension in Hindi. This makes it unlikely that our findings are caused by the split character of Hindi.

Nevertheless, we consider the extension of this work to languages with consistently ergative case marking an important objective for future research. In its strongest form, the S/A preference hypothesis predicts that even in non-split ergative languages, base-form initial NPs will transiently receive an S or A interpretation during incremental processing, despite the fact that such an NP must invariably be interpreted as encoding a P argument at the end of the sentence. Alternatively, the S/A preference could go only so far as base-form NPs at least have the potential for role ambiguity. In this case one would predict that the processing system adapts to the specific affordances of consistently ergative case-marking systems and completely refrains from assigning A roles to base-form NPs, or does this only to a much smaller extent (yielding weaker ERP effects).

Finally, it appears important to consider possible consequences of the fact that our participants were not monolingual speakers of Hindi and, accordingly, spoke at least one (English) or possibly multiple languages with non-ergative case-marking systems. While we acknowledge that this may have impacted upon our results, it may prove to be a general limitation for language processing studies on ergative languages. For the majority of languages with ergative case marking that are accessible to psycholinguistic and neurolinguistic research, speakers will likely have some knowledge of a language without ergative case marking (e.g. Basque speakers’ knowledge of Spanish, speakers of languages of the Caucasus’ knowledge of Russian, speakers of indigenous Australian languages’ knowledge of English etc.). Moreover, with multilingualism from early childhood a reality in many parts of the world outside of the mainstream Western cultural sphere, any attempts at examining language processing in typologically diverse languages will necessarily be confronted with this issue. A highly pertinent goal for future research should thus be to consider how to deal with the fact that the monolingual native speaker is but an idealization in many parts of the world. Importantly, however, our results suggest that when speakers are proficient in multiple systems, the S/A preference appears to be detectable in sentence processing.

## Evidence from Language Evolution

### Materials and Methods

#### Data

Data on case marking was extracted from available grammars and entered into a database [S1 Database and S1 Sources], keyed to two genealogical language classifications, the autotyp [52] and the glottolog [53] taxonomies. We sampled language families as densely as possible. Especially for small families, we attempted to include all information that we could find so as to achieve the best possible estimates on bias towards or against ergatives in the evolution of each family.

We limited our survey to default verb classes, defined as the largest class in the lexicon of a language that has no further specifications or constraints. Verbs with non-default case frames (e.g. Russian verbs assigning dative rather than accusative case to objects) were excluded from the data because the S/A preference in processing holds only for verbs of default classes. Verbs of non-default classes do not show the preference. For example, because in Hindi a few non-default verbs assign the accusative marker *ko* to S and A roles, Hindi NPs marked by *ko* cover S, A and P roles alike. However, despite the fact that such a distribution is identical to the distribution of base-form NPs across roles, *ko*-marked NPs do not show an S/A preference effect [54]. Similar observations hold for German [55] and Japanese [56], and we suspect that this is universally so.

When languages use ergatives in only some grammatical subsystems, we listed each subsystem separately in the database and analyzed each as one possible evolutionary descendant of an ancestor language (i.e. as an independent evolutionary trial), in line with standard assumptions that language change operates on individual items rather than on entire grammar systems. For example, in Hindi, ergative marking occurs in perfective main clauses, but never in dependent clauses. From this observation we conclude that two conditions were relevant for the evolution of case in Hindi. Accordingly, we take ‘perfective + main’, ‘imperfective + main’, ‘perfective + dependent’ and ‘imperfective + dependent’ as separate evolutionary trials and ask in each of the four cases whether or not the language developed or maintained ergative marking. However, there is one point in the analyses where we treated such subsystems not as independent evolutionary trials but as uncertainties in the system, i.e. as polymorphisms: this was when performing transition rate estimates on lexically-based phylogenetic trees because these trees necessarily have entire language systems at their tips (see the Methods section below).

When there was no evidence of a split into subsystems, we assumed that there was only one evolutionary trial, for the whole language. Any alternative to this would have to make a guess as to what kinds of subsystem distinctions might have been relevant in the past but never had an effect. This requires speculation well beyond evidence.

The database contains 705 data-points from 617 languages, classified in 156 autotyp families and in 144 glottolog families. Of these families, 80 families have only one member, i.e. represent isolate languages with no relatives in our database, or indeed without any known relatives at all (such as Basque).

In order to control for possible local counter-biases towards ergatives in the Pacific region and in South America resulting from language contact [Fig 1], we classified the data into five large geo-linguistic areas. The boundaries of these follow conventional continent boundaries. As the boundary between Eurasia and the Pacific region (New Guinea, Australia, Oceania) area, we chose the Wallace Line [52]. However, shifting this boundary further west or east has no impact on results because the insular Southeast Asia region is heavily dominated by families lacking ergatives anyway [Fig 1].

The Austronesian and Semitic families in our database straddle two areas of interest: Eurasia and the Pacific region in the case of Austronesian; Eurasia and Africa in the case of Semitic. In order to explore family biases separately within each area (see Methods), we split these families into smaller sub-families (subgroups) that each fall completely within one of the areas. This increases the number of families available for testing. In one case, this resulted in a large sub-family: Oceanic in the autotyp taxonomy (*N* = 18), and Central-Eastern Malayo-Polynesian subgroup in the glottolog taxonomy (which is rejected as a proven clade in the autotyp taxonomy) (*N* = 20). In all other cases, the resulting sub-families have less than five members.

#### Data availability

The database used in the analysis, including references for the original analyses in each language, is available as S1 Database and S1 Sources in the Supporting Information.

#### Methods

Estimating directed biases, i.e. evolutionary preferences for or against ergatives in the historical evolution of languages must be based on the results in extant languages because apart from very few exceptions, we have no data from ancestral stages. We estimate directed biases in large families (*N* ≥ 5) in Step 1, and then, in Step 2, extrapolate to smaller and single-member families [11, 57]. (The cut-off point between large and small families was chosen so that methods performed reasonably well, and that there is a balance between the two sets; we found no evidence that changing the cut-off point changes the overall results).

**Step 1** For large families, we use two methods, each applied to the two taxonomies. One method, the *set-based method*, relies on binomial tests within each family and interprets a significant majority value (e.g. for ergatives) as reflecting a directed bias for that value in the history of the family, either because the family had this value from the beginning (e.g. the proto-language was ergative) and preferentially retained it, or because the value was innovated early or repeatedly, or both [57]. This method makes no commitment as to whether languages developed through trees or through dialectological waves or cascades, but rather treats each value in the tips of the family as resulting from an independent binomial trial in the history of the family. Also, the method requires no information on branch lengths in the tree. The method is implemented as an R package [58], applied here with default settings.

The second method, called here *tree-based method*, estimates the transition rate matrix of a Continuous-Time Markov model, using either a Maximum Likelihood (ML) or a Bayesian Markov Chain Monte Carlo (MCMC) approach, and then evaluates the relative evidence for a directed bias, i.e. strongly unequal transition rates vs. equal rates on the basis of likelihood ratio tests with an *α* = .05 rejection level (for ML-based models) or Bayes Factor assessments with a logBF > 2 positive evidence criterion (for MCMC-based models) [59, 60]. This method is only applicable to families with some variation (i.e. it cannot be applied to families in which all languages are fully ergative or fully non-ergative), assumes a tree-based model of language evolution, and requires branch length estimates in the tree. Branch lengths are not contained in the autotyp and glottolog taxonomies. In response to this, we set branch lengths to 1 between each node in the tree, assuming that each major type change (such as between ergative and non-ergative case marking) requires the birth of a new language, i.e. a node, and assuming that rates of non-cladogenetic change elsewhere in the language (e.g. change in lexical cognate replacement) have no impact on the number of opportunities for type change in case marking. These assumptions are consistent with the research tradition in historical linguistics as well as with the more recent observation that structural change tends to be characterized by punctuated evolution [61].

Nevertheless, for two families, Austronesian and Indo-European, we had access to branch length estimates derived from Bayesian phylogenetic analyses of lexical data [9], and so we compared transition rates between ergative and non-ergative markers in these trees as well. However, the lexically-based trees had to be severely pruned due to differences in the language samples, and so transition rates estimates were based on only 15 and 22 languages in Austronesian and Indo-European, respectively.

For MCMC models, we used the software package BayesTraits, version 2 [62], whereas for ML models, we used both BayesTraits and fitDiscrete in the R package geiger [63].

**Step 2** Evolutionary biases behind smaller families (with 1 to 4 representatives) are estimated via extrapolation techniques (described and justified in detail in [57] and implemented as an R package in [58]): we first estimate the probability of a bias in any direction, *Pr*(bias), in large families within each geo-linguistic area. For this, we use Laplace’s Rule of Succession applied to the bias estimates from Step 1, so as to avoid unrealistic *Pr*(bias) = 1. We then draw random samples of small families from a binomial distribution with *Pr*(bias) and declare them as the sole survivors of larger unknown families with a bias. Some of these survivors can be assumed to directly represent the bias of the unknown larger family, and so we can take their structural choice (e.g. having an ergative, as in Basque) to reflect the direction of the bias in the family (picking the majority value if there is more than one survivor, or making a random choice in the case of ties). However, some survivors might be deviates, e.g. exhibiting an ergative although the family as a whole was biased against this. We estimate the probability of deviation, *Pr*(deviation), from the strength of the bias in large families within each area (as per Step 1); for instance, a mean 90% bias in large families would yield *Pr*(deviation) = .1. Then, we again draw random samples of deviating members from a binomial distribution with *Pr*(deviation). The resulting samples of deviates are assigned the opposite of their actual value.

The two cases of random sampling (with *Pr*(bias) and *Pr*(deviation)) during these extrapolations incur an error, but the error follows a normal distribution. We therefore performed the extrapolations 10,000 times and then use the mean of these extrapolations as our estimate of bias directions. This results in a general estimate of how many families have undergone a history that was biased towards the development and maintenance of ergatives and of how many have undergone a history biased against the development and maintenance of ergatives.

These count estimates were finally subjected to log-linear modeling with likelihood-ratio tests assessing the significance of the factors bias direction (favoring vs. disfavoring ergatives) and geographical area. Families with no signal of any evolutionary bias do not give evidence for or against our prediction because the distributions in these families are compatible both with incipient developments towards ergatives and away from ergatives. We therefore exclude these families from the log-linear models.

Results are visualized using mosaic plots using the R package vcd [64]. The full R script (including the results of each step of analysis) is available as S1 Script in the Supporting Information.

### Results

Table 2 summarizes the mean estimates of how many families have an evolutionary bias towards (‘E’) or against ergatives (‘A’), comparing the results from the two taxonomies (autotyp and glottolog) and the three methods for estimating biases in large families (set-based, tree-based with ML, tree-based with MCMC estimates). The count estimates in the table are based on 10,000 extrapolations each, and this never led to standard errors exceeding.03. The bias and deviation probabilities that formed the basis for the extrapolations are reported in detail in S2 and S3 Tables.

**Table 2:** Estimated counts of families with an evolutionary bias towards vs. against ergatives, across methods, taxonomies and areas.

Area	Bias	autotyp		glottolog	
		Binom./MCMC	ML	Binom./MCMC	ML
Africa	none	4.17	1.26	3.27	3.27
Africa	A	34.83	28.74	41.73	41.73
Africa	E	0.00	0.00	0.00	0.00
Eurasia	none	1.29	8.49	7.30	7.32
Eurasia	A	28.71	24.12	23.69	23.67
Eurasia	E	0.00	3.39	3.01	3.01
Pacific	none	8.50	30.21	15.00	14.98
Pacific	A	24.11	14.02	21.14	21.23
Pacific	E	3.40	3.78	5.86	5.79
South America	none	12.00	12.01	12.47	12.48
South America	A	9.99	9.98	10.03	10.00
South America	E	2.01	2.01	2.50	2.52
Rest of the Americas	none	24.03	4.12	4.27	4.23
Rest of the Americas	A	18.76	34.88	35.73	35.77
Rest of the Americas	E	5.21	0.00	0.00	0.00

The table reports the means of 10,000 extrapolations. The standard errors of these are all smaller than.03. The extrapolations are based on large-family estimates of biases using set-based methods (with binomial tests) and tree-based methods (using MCMC and ML techniques, as described in the Methods section). MCMC-methods fully converged with binomial test results, resulting in the same estimates of counts. The two variants of ML-methods used here (fitDiscrete and BayesTraits) agreed among themselves but resulted in slightly different total estimates compared to the set-based and MCMC-based counts. *Codes:* A, against ergatives; E, towards ergatives.

**Fig 5:**
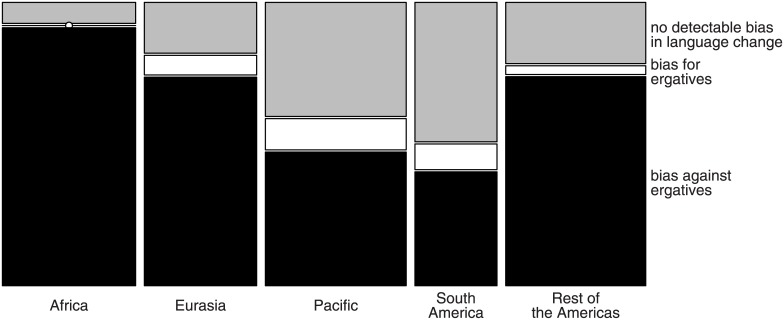
Grand-average of estimated evolutionary biases within families, across methods and taxonomies. In each area, there are significantly more families that are estimated to be biased against the development or maintenance of ergatives (black tiles) than there are families that are estimated to be biased towards ergatives (white). Families without a bias (grey) provide no evidence on evolutionary biases in language change because they are compatible both with incipient developments towards ergatives or away from ergatives. *Note:* The sizes of tiles in the figure are proportional to the frequency of families tested (total *N* = 182).

Table 3 shows the result of the statistical analysis of the estimated counts. The interaction between geographical area and bias direction were significant in all cases and was resolved by binomial tests within each area (reported in the last column of Table 3). The results show that while there are significant differences between areas, families with biases against ergatives always significantly outnumber families with biases towards ergatives. Results converged across the two taxonomies and the three estimation methods. Fig 5 summarizes the grand-average of the results.

**Table 3:** Analysis of bias directions, separate for each taxonomy and method.

Taxonomy	Method	LR test of interaction *χ* ^2^(4), *p*	LR test of bias *χ* ^2^(5), *p*	Binomial tests per area All *p*s
autotyp	Binom./MCMC	15.81, .003	121.84, < .001	< .02
autotyp	ML	15.37, .004	119.01, < .001	< .02
glottolog	Binom./MCMC	21.30, < .001	139.53, < .001	<. 05
glottolog	ML	21.30, < .001	139.53, < .001	< .05

Likelihood ratio (LR) tests compare loglinear models with vs. without an interaction term (area × bias direction, third column) and with vs. without the bias direction term (fourth column). Binomial tests were performed separately for each area in order to resolve the interaction (last column).

The convergence across methods and taxonomies can be traced back to the fact that only two autotyp and one glottolog family showed differing results across methods and taxonomies in the large family bias estimates (Step 1, as described in the Methods section). For these families, the set-based and the MCMC-based methods, but not the ML method, suggested biases against ergatives [S4 and S5 Tables]. The lexically-based Austronesian and Indo-European trees [9] converged with the set-based and MCMC-based methods, with logBF = 5.263 and logBF = 6.495 evidence for biases against ergatives, respectively.

None of the large families revealed a bias towards ergatives. The estimated counts of such biases in Table 2 all stem from the extrapolation to small families and isolates.

### Discussion

The results of our study suggest that languages tend to avoid ergatives when they evolve over time: if a language has ergative case marking, it is more likely to lose than to keep it, and if a language lacks ergative case marking, it is unlikely to develop it. To be sure, ergative cases can arise and be maintained for a while, but the probabilities of this are always lower than the probabilities of avoiding ergatives. The probabilities for developing and maintaining ergative case marking are a bit higher in the Pacific and in South America than in other regions, but they never approach the probabilities for families to develop away from ergatives. The observed variation in probabilities is likely to reflect a multitude of factors, from language contact that favors ergative case marking if contact languages also have ergatives [65], to random fluctuations stemming from the uncertainty at which a possible language change (e.g. reanalysis of a base-form NP as covering S and A, reducing ergatives) is indeed actualized in a given group of speakers.

A more principled scenario that could work against the general trend is conceivable if a language is consistently ergative. In such a language, base-form NPs would never allow reference to an A argument, making a historical reanalysis towards an interpretation as S or A difficult. Indeed, such a language could in principle even train the comprehension system not to assign an A role at any point during incremental processing, making reanalyses even less likely. In the absence of neurophysiological research on such languages, we cannot presently assess this possibility. However, the results from the language evolution study make the possibility not very likely: while there are individual languages that are consistently ergative, this is never an observable evolutionary bias in any extant large family. Thus, although consistent ergativity is a possible escape from the pressure of the S/A processing preference, it is an option that appears to be very rarely taken.

## General Conclusions

The neurophysiological data on Hindi show that the S/A preference persists even if (i) a language contains structures that directly contradict the needs of the processing system by having base-forms that separate the marking of A from the marking of S arguments, (ii) initial NPs are inanimate and therefore unlikely agents, and (iii) initial NPs are typically patients because S and A arguments are often dropped. This supports the theory that the S/A preference is a species-wide property of the processing system which is independent of individual languages or contexts, and does not adapt to the affordances of these languages or contexts. As such, the S/A preference is expected to exert systematic pressure on languages to favor non-ergative case systems that treat S and A roles alike over ergative case systems that separate the marking of S from that of A roles. The results from our estimates on evolutionary biases support this hypothesis. Languages are more likely to loose than to gain ergative case marking.

In conclusion, we find that one specific property of the human language processor, the S/A-preference and ultimately a drive for simplicity, causes languages to disfavor a certain type of grammar, one with an ergative marker, when they evolve over time.

## Supporting Information

S1 Database Database of case-marking patterns. (CSV)

S1 Sources Bibliographical references of the sources for the S1 Database. (BIB)

S1 Script Script and step-by-step results of the language evolution analysis. (R)

S1 Table Additional sample set of materials from the electrophysiological experiment on Hindi: masculine P arguments and feminine A arguments. (PDF)

S2 Table Laplace bias estimates of *Pr*(bias in any direction) in large families (*N* ≥ 5) across methods and taxonomies. (PDF)

S3 Table Estimated probability *Pr*(deviation) in small families. (PDF)

S4 Table Comparison of bias estimates across methods in large families with variation (i.e. not all ergative or all non-ergative), using autotyp taxonomies. (PDF)

S5 Table Comparison of bias estimates across methods in large families with variation (i.e. not all ergative or all non-ergative), using glottolog taxonomies. (PDF)
