# Supplementary material for: The Neurophysiology of Language Processing Shapes the Evolution of Grammar: Evidence from Case Marking
Source: PLoS One. 2015 Aug 12;10(8):e0132819. doi: 10.1371/journal.pone.0132819 (PMC4534460; doi:10.1371/journal.pone.0132819)
Supplement: S1 Table — (PDF) [file pone.0132819.s004.pdf]

S1 Table. Additional sample set of materials from the electrophysiological experiment on Hindi: masculine P-arguments and feminine A-Arguments

Condition	Examples
AI	*pānī* *pī-tī* *hai* *Anitā*<br>water(M)[NOM] drink-I.F AUX Anita(F)[NOM]<br>'Anita drinks water.'
UI	*pānī-ko* *pī-tī* *hai* *Anitā*<br>water(M)-ACC drink-I.F AUX Anita(F)[NOM]<br>'Anita drinks water.'
AP	*pānī* *pī-yā* *hai* *Anitā-ne*<br>water(M)[NOM] drink-P.M AUX Anita(F)-ERG<br>'Anita has drunk water.'
UP	*pānī-ko* *pī-yā* *hai* *Anitā-ne*<br>water(M)-ACC drink-P.M AUX Anita(F)-ERG<br>'Anita has drunk water.'

Condition codes: A, ambiguous; U, unambiguous; I, imperfective aspect (not triggering ergative marking); P, perfective (triggering ergative marking). *Glosses:* AUX, auxiliary; ACC, accusative; ERG, ergative; F, feminine; M, masculine; NOM, nominative.
