# Supplementary material for: The Neurophysiology of Language Processing Shapes the Evolution of Grammar: Evidence from Case Marking
Source: PLoS One. 2015 Aug 12;10(8):e0132819. doi: 10.1371/journal.pone.0132819 (PMC4534460; doi:10.1371/journal.pone.0132819)
Supplement: S2 Table — (PDF) [file pone.0132819.s005.pdf]

S2 Table. Laplace bias estimates Pr(bias in any direction) in large families ($N \geq 5$) across methods and taxonomies.

	AUTOTYP		GLOTTOLOG	
	Binom./MCMC	ML	Binom./MCMC	ML
Africa	0.93 (0.75, 1)	0.93 (0.75, 1)	0.91 (0.69, 1)	0.91 (0.69, 1)
Eurasia	0.75 (0.48, 0.94)	0.75 (0.48, 0.94)	0.77 (0.52, 0.95)	0.77 (0.52, 0.95)
Pacific	0.5 (0.27, 0.73)	0.38 (0.16, 0.62)	0.64 (0.35, 0.88)	0.64 (0.35, 0.88)
South America	0.5 (0.15, 0.85)	0.5 (0.15, 0.85)	0.5 (0.15, 0.85)	0.5 (0.15, 0.85)
Rest of the Americas	0.88 (0.59, 1)	0.88 (0.59, 1)	0.88 (0.59, 1)	0.88 (0.59, 1)

95% credibility intervals in brackets.
