# Supplementary material for: The Neurophysiology of Language Processing Shapes the Evolution of Grammar: Evidence from Case Marking
Source: PLoS One. 2015 Aug 12;10(8):e0132819. doi: 10.1371/journal.pone.0132819 (PMC4534460; doi:10.1371/journal.pone.0132819)
Supplement: S3 Table — (PDF) [file pone.0132819.s006.pdf]

S3 Table. Estimated probability Pr(deviation) in small families.

	AUTOTYP		GLOTTOLOG	
	Binom./MCMC	ML	Binom./MCMC	ML
Africa	0.00	0.00	0.00	0.00
Eurasia	0.03	0.03	0.03	0.03
Pacific	0.03	0.00	0.07	0.06
South America	0.00	0.00	0.00	0.00
Rest of the Americas	0.00	0.00	0.00	0.00

Pr(deviation) is the probability that the sole survivor(s) of a larger unknown family that is sampled with Pr(bias) as exhibiting a bias is in fact not representative of the direction of that bias, i.e. exhibits a structure that deviates from the bias that characterized the larger family.
