# Supplementary material for: The Neurophysiology of Language Processing Shapes the Evolution of Grammar: Evidence from Case Marking
Source: PLoS One. 2015 Aug 12;10(8):e0132819. doi: 10.1371/journal.pone.0132819 (PMC4534460; doi:10.1371/journal.pone.0132819)
Supplement: S4 Table — (PDF) [file pone.0132819.s007.pdf]

S4 Table. Comparison of bias estimates across methods in large families with variation (i.e. not all ergative or all non-ergative), using AUTOTYP taxonomies

	majority value	majority proportion	Set-based	Tree-based		
				ML	ML	MCMC
				`fitDiscrete`	`BayesTraits`	`BayesTraits`
			$P_{\text{Binom. test}}$	$P_{\text{LR test}}$	$P_{\text{LR test}}$	$\log \text{BF}$
Oceanic	A	0.889	0.001	0.311	0.120	8.202
Cariban	none	NA	0.500	0.705	0.705	-0.972
Chimbu-Wahgi	none	NA	0.145	0.434	0.192	0.521
Eastern Highlands	none	NA	0.363	0.259	0.482	-0.502
Finisterre-Huon	none	NA	0.773	0.705	0.705	-0.563
Binanderean	none	NA	0.500	0.654	0.654	-0.625
Gunwingguan	none	NA	0.656	1.000	1.000	-0.947
Indo-European	A	0.770	0.000	0.000	0.000	19.098
Koiarian	none	NA	0.109	0.547	0.198	0.685
Madang	A	0.875	0.035	0.330	0.212	2.475
Nakh-Daghestanian	none	NA	0.868	0.369	0.369	-0.593
Pama-Nyungan	none	NA	0.734	0.617	0.617	-1.149
Pano-Tacanan	none	NA	0.891	0.410	0.410	-0.496
Sino-Tibetan	none	NA	0.944	0.147	0.147	0.525

The results from a Bayes Factor analysis of MCMC models of transitions rates agree with the results from a simple binomial tests. The only divergences in the results is found with ML models in the case of the Oceanic and Madang families.
