# Supplementary material for: The Neurophysiology of Language Processing Shapes the Evolution of Grammar: Evidence from Case Marking
Source: PLoS One. 2015 Aug 12;10(8):e0132819. doi: 10.1371/journal.pone.0132819 (PMC4534460; doi:10.1371/journal.pone.0132819)
Supplement: S5 Table — (PDF) [file pone.0132819.s008.pdf]

S5 Table. Comparison of bias estimates across methods in large families with variation (i.e. not all ergative or all non-ergative), using GLOTTOLOG taxonomies

	majority value	majority proportion	Set-based	Tree-based		
				ML `fitDiscrete`	ML `BayesTraits`	MCMC `BayesTraits`
			$P_{\text{Binom. test}}$	$P_{\text{LR test}}$	$P_{\text{LR test}}$	log BF
CE Malayo-Polynesian	A	0.900	0.000	0.181	0.076	10.836
Cariban	none	NA	0.500	0.705	0.705	-0.718
Gunwinyguan	none	NA	0.656	1.000	1.000	-0.875
Indo-European	A	0.773	0.000	0.000	0.000	21.374
Koiarian	none	NA	0.109	0.418	0.164	0.917
Nakh-Daghestanian	none	NA	0.868	0.369	0.369	-0.422
Nuclear Trans New Guinea	A	0.682	0.011	0.028	0.029	3.570
Pama-Nyungan	none	NA	0.734	0.617	0.617	-1.524
Panoan	none	NA	0.813	0.654	0.654	-0.579
Sino-Tibetan	none	NA	0.944	0.147	0.147	0.141

The results from a Bayes Factor analysis of MCMC models of transitions rates agree with the results from a simple binomial tests. The only divergence in the results is found with ML models in the case of the Central-Eastern Malayo-Polynesian families.
